# Defined Nanoscale Chemistry Influences Delivery of Peptido-Toxins for Cancer Therapy

**DOI:** 10.1371/journal.pone.0125908

**Published:** 2015-06-01

**Authors:** Santosh K. Misra, Mao Ye, Sumin Kim, Dipanjan Pan

**Affiliations:** Department of Bioengineering, University of Illinois at Urbana-Champaign, Urbana, IL, 61801, United States of America; Beckman Institute of Advanced Science and Technology, University of Illinois at Urbana-Champaign, Urbana, IL, 61801, United States of America; Department of Materials Science and Engineering University of Illinois at Urbana-Champaign, Urbana, IL, 61801, United States of America; Carle Foundation Hospital, Urbana, IL, 61801, United States of America; The City University of New York-Graduate Center, UNITED STATES

## Abstract

We present an *in-silico-to-in-vitro* approach to develop well-defined, self-assembled, rigid-cored polymeric (Polybee) nano-architecture for controlled delivery of a key component of bee venom, melittin. A competitive formulation with lipid-encapsulated (Lipobee) rigid cored micelle is also synthesized. In a series of sequential experiments, we show how nanoscale chemistry influences the delivery of venom toxins for cancer regression and help evade systemic disintegrity and cellular noxiousness. A relatively weaker association of melittin in the case of lipid-based nanoparticles is compared to the polymeric particles revealed by energy minimization and docking studies, which are supported by biophysical studies. For the first time, the authors’ experiment results indicate that melittin can play a significant role in DNA association-dissociation processes, which may be a plausible route for their anticancer activity.

## Introduction

Host defense peptides (HDPs) are a class of evolutionarily conserved substances of the innate immune response that are recognized as chief players in the defense system found among all classes of life. They are usually amphipathic, have a net positive charge (generally +2 to +9) and are short in sequence (10–100 aa); furthermore, HDPs have recently been explored for their anticancer property [1–4]. This class of peptides features many characteristics ideal for anticancer treatment applications, such as i) high water solubility, ii) a broad spectrum of cytotoxicity, and iii) the ability to overcome multidrug resistance, which has developed in cancer cells treated with conventional chemotherapy drugs [5]. Several biophysical studies have shown that small proteins or peptides (20–40 amino acid residues) can penetrate the cell membranes of microorganisms. Melittin, a cationic amphipathic peptide made up of 26 amino acid (aa) residues, has been found to be a potent component of bee venom *Apis mellifera* [6]. It has been proven to have a direct cytotoxic effect on a wide range of cancer cell lines *in vitro*. It has been reported that melittin inhibited cell growth in two ovarian cancer cells via induction of death receptors and down regulation of JAK2/STAT3 [7, 8]. It exerts its toxic activity by disrupting plasma membranes following pore formation. Cationic aa residues of melittin interact directly with anionic cellular membranes via electrostatic interactions and hydrophobic regions; this interaction is responsible for membrane permeation and disruption [6]. A comparably short protein, with an end-to-end distance of ~3.5 nm, the dimension perfectly serves as a single transmembrane-spanning alpha-helix. Numerous computational studies have demonstrated that melittin forms transmembrane pores from its interaction with lecithin PC membranes (2~3 nm in diameter). Its potent activity has attracted researchers to utilize melittin for the next generation anticancer therapeutic agent. However, the therapeutic potential has not been fully achieved in clinic due to their off-target toxicity, rapid degradation and clearance *in vivo*. Melittin has been incorporated into lipid coated perfluorocarbon particles to accumulate in multiple tumor targets, dramatically reducing tumor growth [7].

**Fig 1:**
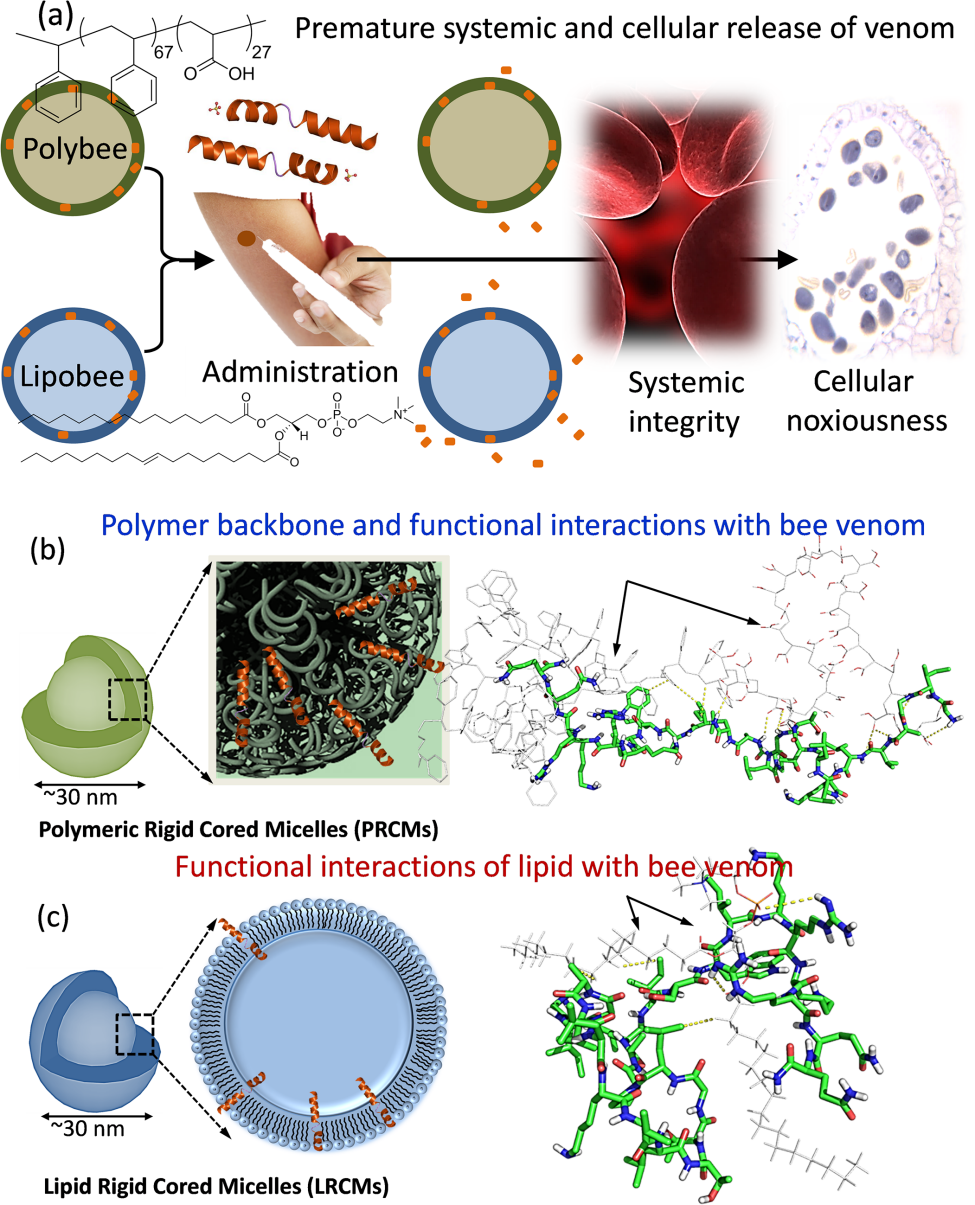
Graphical representation of peptidotoxin delivery. (a) Schematic of administrative protocol for Lipobee and Polybee; (b) Initial docking images of the PS_67_-*b*-PAA_27_ and melittin systems showing how structures of melittin are bound to single amphiphilic polymers and (c) showing the docking structure of melittin and lecithin PC. PS_67_-*b*-PAA_27_ and lecithin are depicted by white lines with explicit oxygen atoms depicted in red. The melittin peptide is shown in a green chain link style with oxygen atoms depicted as red and nitrogen depicted as blue.

Although a few of these approaches clearly promise impending success in preclinical studies, their translational potential has not been fully realized. None or very little information can be found in the literature regarding their translational use in human studies. To improve selectivity and reduce toxicity, delivery vehicle implementation in human subjects will require great care. It is, therefore, imperative that we emphasize the fundamental chemical strategy and rationally approach the design of the vehicle suited for translational use. A better understanding of the interaction of venom toxins at the nanoscale is critical, which may dictate its overall stability, systemic integrity and cellular noxiousness. A carefully structured study to comprehend the interactions of melittin with the functional components at the shell and shell-surface will drive the design of next-generation delivery vehicles. Towards this end, we have adopted an in-silico-to-in-vitro approach and developed a well-defined nanoparticulate system for controlled delivery of melittin. The goal of this work was to provide a rational nanoparticle-based design for venom delivery through computational studies and support our theoretical findings with physico-chemical and biological studies. Thus, following the syntheses and physico-chemical characterization, a series of sequential experiments were carried out to study how nanoscale chemistry influences the delivery of venom toxins for cancer regression and help evade systemic disintegrity and cellular noxiousness (Fig 1).

*In silico* studies revealed the higher stability response of melittin towards amphiphilic block polymers compared to lipid molecules. Experimental study confirmed the better stability of polymeric system over lipidic assembly. To introduce micellar stability, a concept of rigid core was introduced [9]. Studies exploring change in hydrated size and inertness against serum proteins revealed the higher stability of rigid core particles. Experiments on melittin leaching in the presence of serum concentration revealed the higher stability of a melittin-polymer system (Polybee) compared to a melittin-lipid (Lipobee) system. An in silico study on melittin-DNA interaction was performed and verified by experimental data. It was found that free melittin could bring significant change in inter-helix hydrogen bonding to potentially influence cell growth mechanisms. Melittin in its protected form as Polybee and Lipobee were inactive. Significant changes in the hydrated size of Polybee and Lipobee upon incubation with sodium dodecyl sulfate was observed but not a lower pH. This pointed to the anionic membrane interaction as the responsible factor inside the cytoplasm as a plausible melittin release mechanism. Breast cancer cells of a different estrogen receptor status were used as model *in vitro* cancers for growth inhibitions studies. Irrespective of the cell line, Polybees were found to be better anti-cancer formulations compared to Lipobee and free melittin control.

## Results and Discussion

To design a safer as well as efficacious delivery system, we pursued a rigid core nanosystem that can potentially retain their integrity in blood circulation following systemic administration [10a-c]. At the nanoscale level, rigid core micellar (RCM) systems can either be stabilized by amphiphilic PS_67_-*b*-PAA_27_ (polystyrene-b-polyacrylic acid) (PRCM) or by phospholipids (lecithin PC) (LRCM) encapsulation (Fig 1A). We anticipate that this system will provide model architectures, since the majority of nanomedicine platforms are dominated by lipid and polymeric systems. Furthermore, this strategy can also be extended to a series of peptide-toxins of different natural sources, chemistries and sizes.

**Fig 2:**
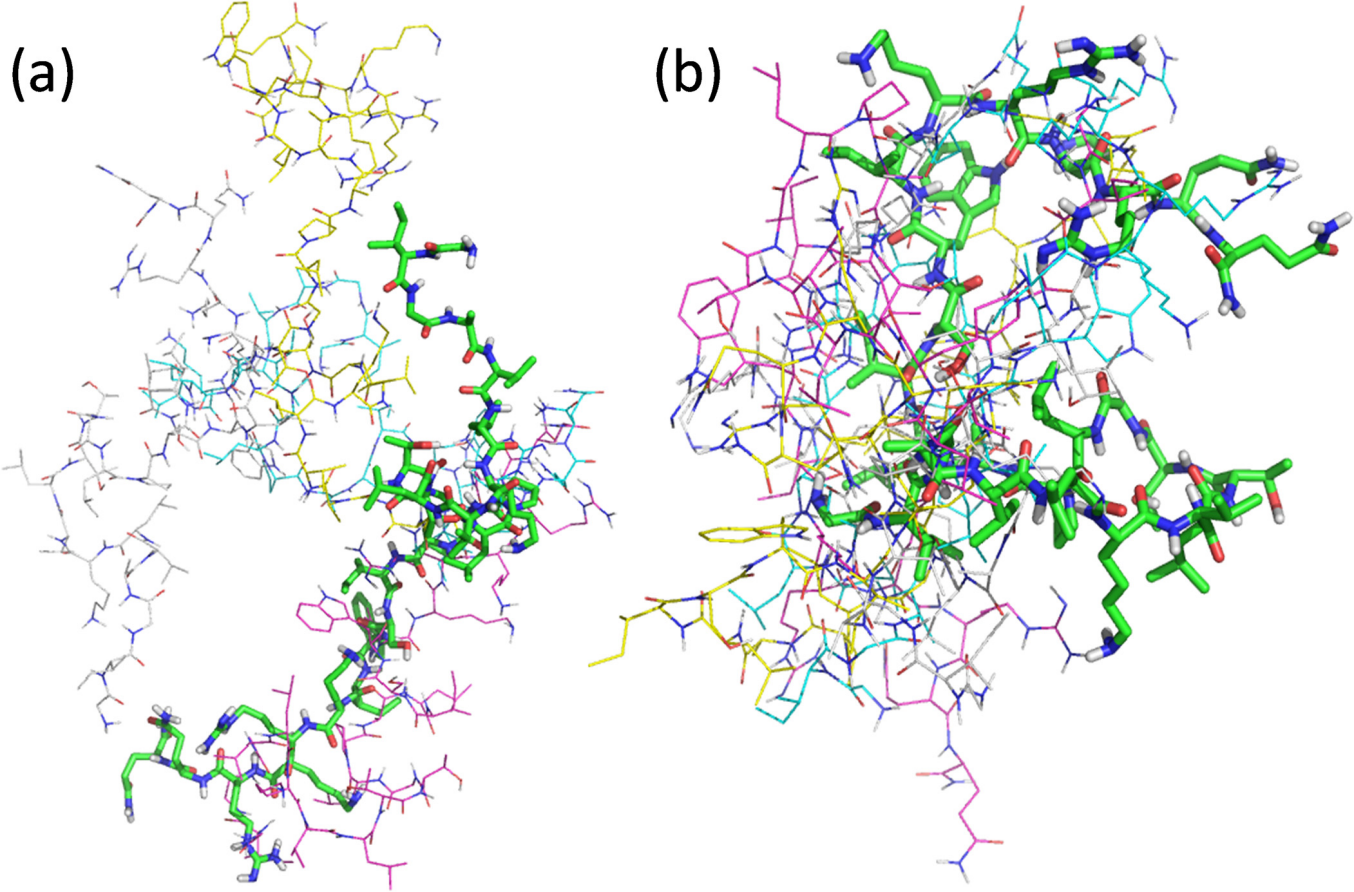
Molecular docking studies. Super-imposition of the five best docking poses of melittin with lecithin PC and PS_67_-*b*-PAA_27_ polymer: (a) Docking poses of melittin to PS_67_-*b*-PAA_27_ polymer; (b) docking poses of melittin to lecithin PC. The best scored pose is in the green linked chains with the following smaller attachments listed in order according to their score as indicated by their color: Magenta, 2nd; yellow, 3rd; white, 4th, and cyan, 5th.

To investigate the key interactions in the melittin- PS_67_-*b*-PAA_27_ polymer and melittin-lecithin PC lipid systems, molecular docking simulations were performed and analyzed (Fig 1B and 1C). All molecules were minimized (Sybyl-X 2.0) [11] before docking with MOE 2013.08. To investigate the key interactions in the melittin- PS_67_-*b*-PAA_27_ polymer and melittin-lecithin PC lipid system, MOE-Dock [12] was employed to dock the minimized melittin to both PS_67_-*b*-PAA_27_ polymer and lecithin PC lipid systems. The five best docking poses with highest S scores (lowest docking energy) were retained and listed in tabular form. Superimpositions of the five best docking poses in both systems are showed in Fig 2. From Fig 2, it can be found that in melittin- PS_67-_ *b*-PAA_27_ polymer docking structure, the five docking poses are in great diversity, which results in the great difference in the docking score between pose 1 and pose 5 (14.5 kcal/mol). However, in melittin-lecithin PC lipid docking structure, five docking poses can superimpose much better, which resulted in a small docking score difference between pose1 and pose 5 (1 kcal/mol). The conformation differences of docking poses of melittin to PS_67_-*b*-PAA_27_ polymer and lecithin PC lipid was caused by the different electric fields and steric fields for these two systems.

Fig 1**A** shows the docking structures of best poses of melittin to PS_67_-*b*-PAA_27_ polymer (Fig 1B) and lecithin PC lipid system (Fig 1C). In Fig 1B, it can be seen that melittin peptide stays close and parallels well with the hydrophilic end and a part of hydrophobic section of the polymer. The hydrophilic acrylic acid residues of the polymer formed critical hydrogen bond interactions with amino and hydroxyl groups of aa residues. Hydrophobic phenyl moiety near the hydrophilic terminus of the polymer formed hydrophobic interactions with side chains of aa residues of melittin. Gly1 to Ile17 lie in the hydrophilic end whereas Ser18 to Gln26 lie in the hydrophobic end of the polymer. In detail, the amino groups of the backbone of Gly1, Gly3, Ala4, Val5, Leu6, Gly12, Ala15 and Ile17, the oxygen both in side chain and backbone of Thr11 formed hydrogen bond interactions with oxygen of carboxylic acid of the polymer. The side chains of Leu17, Trp19, Lys21, Arg24 and Gln26 form hydrophobic interactions with phenyl and carbon atoms of the backbone of the polymer. To compare the key interactions of the docking poses with lecithin PC-peptide model, we docked the melittin peptide to the lipid lecithin PC (Fig 1C). Though melittin was found to be well intertwined with the lipid, this peptide-lipid docking structure was much unfastened. The distance between the two molecules was not close enough; therefore, not many hydrogen bond interactions and hydrophobic interactions exist as observed for peptide-polymer complex. The only interactions identified were the amino groups in Arg22 and Arg24 forming hydrogen bond interactions with oxygen in keto and phosphono groups in lecithin PC lipid. The side chains of Ala4, Leu13 and Ile17 formed hydrophobic interactions with the alkyl group in the lipid.

To further explore the sequence and size dependence of peptides for Polybee and Lipobee carriers, we chose several melittin peptidic fragments for computational modeling studies (S1 Fig). We selected (1) right 17-residues peptide, (2) left 9-residues peptide, and (3) middle 16-residues peptide to dock to PS_67_-*b*-PAA_27_ polymer and lecithin PC lipid system, respectively. In the docking structure of right 17-residues peptide with PS_67_-*b*-PAA_27_ polymer, the amino groups of the backbone of Thr1, the side chain of Lys12, Arg13 and Arg15, the oxygen carbonyl group of Lys12, Gln17 and side chain of hydroxyl group of Thr1, Thr2 and Ser10 formed hydrogen bond interactions with oxygen of carboxylic acid of the polymer. In the lecithin PC lipid docking structure, the amino groups of the backbone of Lys14 and side chain of Arg13, and side chain of hydroxyl group of Thr1 formed hydrogen bond interactions with oxygen in phosphono groups in lecithin PC lipid. In the docking structure of left 9-residues peptide with PS_67_-*b*-PAA_27_ polymer, the amino groups of the backbone of Gly1, Ile2, Ala4 and Leu9, the oxygen of carbonyl group of Val8 formed hydrogen bond interactions with oxygen of carboxylic acid of the polymer. In the lecithin PC lipid docking structure, the amino groups of the backbone of Gly1, Ile2 and the oxygen of carbonyl group of Ile2 formed hydrogen bond interactions with oxygen in phosphono groups in lecithin PC lipid. In the docking structure of the middle 16-residues peptide with PS_67_-*b*-PAA_27_ polymer, the amino groups of the backbone of Thr5, Gly7, Lys16, the side chain of Trp14, the oxygen of carbonyl group of Gly7 and side chain of the hydroxyl group of Ser13 formed hydrogen bond interactions with oxygen of carboxylic acid of the polymer. In the lecithin PC lipid docking structure, the amino groups of the backbone of Leu1, Lys2, Val3, Thr5 and the side chain of hydroxyl group of Thr5 formed hydrogen bond interactions with oxygen in phosphono groups in lecithin PC lipid.

The analysis of docking poses can clearly explain why the peptide-polymer structure is more stable than the peptide-lipid structure (Table 1). We noticed that on the contrary to the docking poses and interactions analysis, the docking energy in lecithin PC lipid system is lower than PS_67_-*b*-PAA_27_ polymer system. This might be caused by differences in average entropy loss/gain due to the conformational flexibility and desolvation energy of each atom rather than that of maximum energy of H-bond between PS_67_-*b*-PAA_27_ polymer and lecithin PC lipid whose sizes are greatly different. In the PS_67_-*b*-PAA_27_ polymer system, the hydrophilic acrylic acid and hydrophobic styrene unit ratio will affect the hydrogen bond and hydrophobic interactions between melittin and polymer systems, whereas the unit length does not change these interactions greatly. S2 Table shows the scores of docking results of three melittin fragments to polybee and lipobee. As evident, the longer the peptide sequences, the better the docking scores were obtained. Docking scores were better for peptide with PS_67_-*b*-PAA_27_ polymer than with lethicin PC lipid because more hydrogen bond interactions are involved in a PS_67_-*b*-PAA_27_ polymer system.

**Table 1:** Scores of different docking poses of melittin superimposed and energy minimized with lipid or amphiphilic polymer system.

Docking Pose	Melittin docked to polymer (Kcal/mol)	Melittin docked to lecithin lipid (Kcal/mol)
Pose 1	-5.07	-8.04
Pose 2	-3.55	-7.51
Pose 3	+2.37	-7.47
Pose 4	+2.81	-7.31
Pose 5	+9.56	-7.14

A post-preparative one-pot insertion method was used for stable entrapment of melittin and a generation of lipidated rigid core micellar melittin (Lipobees) from lipidated rigid core micelles (LRCMs). Similarly, polymerized rigid core micellar melittin (Polybees) were prepared from polymerized rigid core micelles (PRCMs). A typical preparation of LRCMs and PRCMs involved a preparation of ‘rigid core’ of polyoxyethylene20 cetyl ether (PECE) followed by stable coating with lecithin PC or PS_67_-*b*-PAA_27_ [13]. The stability of the micelles was achieved by curing the core at 4°C (PECE mp: 32°C). RCMs were then subjected to post-preparative incubation of the melittin in aqueous suspension for 30 min at ambient temperature with mild vortexing. The hydrodynamic diameter, morphology, layered arrangements; topography, electrophoretic potential, and particle stability were established using various physico-chemical experiments. To find out the loading of melittin in LRCM and PRCM, UV-absorbance spectroscopy was performed. It was seen that signature absorbance for melittin at 290 nm dropped down in the case of Lipobee and Polybee, most likely due to the surface internalization of melittin in LRCM and PRCM with post-interaction methodology.

**Fig 3:**
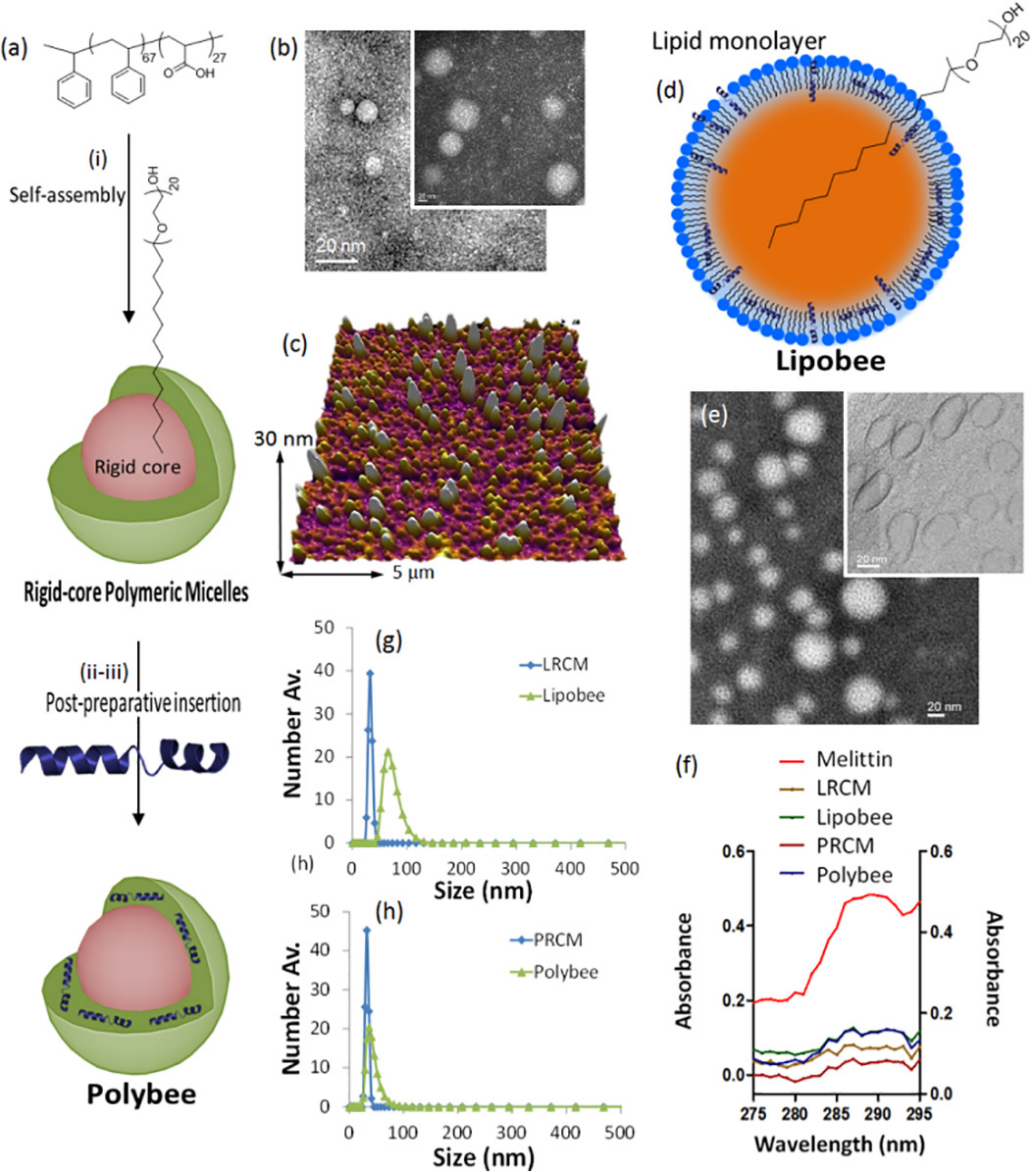
Preparation and physico-chemical characterization studies. Synthesis and characterization of rigid core micelles and melittin loaded particles: (a) Synthesis of PRCM and Polybee nanoparticles; (b) representative TEM images of Polybee; (c) representative AFM images of Polybee; (d) Synthesis of LRCM and Lipobee nanoparticles; (e) representative TEM images of Lipobee; (c) representative AFM images of Lipobee; (f) UV-vis spectroscopy of melittin, LRCM, PRCM, Lipobee and Polybee; (g) hydrodynamic diameter distribution (number averaged, nm). TEM samples (20 μL) were prepared on formvar-coated carbon grids and negatively stained with uranyl acetate and vacuum dried before performing the microscopy. Samples (20 μL) were drop casted on freshly cleaved mica sheets and air dried for >24h before performing the tapping mode AFM.

The LRCM had an average hydrodynamic particle size of 23 ± 2 nm, which grew to 83 ± 3 nm in Lipobee primarily due to the surface interaction of melittin with RCM (Fig 3). Similarly, PRCM showed an average hydrodynamic diameter of 25 ± 5 which increased to 40 ± 8 nm in Polybee (Fig 3). Stability of these PRCM particles across various time points at rt and pH 7.4 was measured using DLS measurements, which showed a nominal change in the size of PRCM and Polybee of less than 10%. Similarly, LRCM size did not change to any significant level while Lipobee showed size changes of ~40%, emphasizing the significant instability of Lipobees compare to the high stability of Polybee. Stability of carrier vehicles has always been major concern in the success of nano-delivery protocols. Hence, Polybee promises the probable better melittin delivery response compare to Lipobee particles during *in vitro* and *in vivo* uses. The surface charge density for PRCMs was -12 ± 1 mV, which dropped down to -6 ± 1 mV in Polybee after incubation with the bee toxins.

On the other hand, zeta potential of LRCMs showed a nominal change in zeta potential when converted to Lipobee. This signifies the efficiency of making Coulombic interactions of the peptide with the outer corona of block polymers comprised of poly(acrylic acid) residues. Anhydrous state morphology of the Lipobee and Polybee particles was obtained at 25 ± 5 nm size compared to 22 ± 6 nm for LRCM and PRCM as studied by transmission electron microscopy (TEM, Fig 3F). The representative atomic force microscopy (AFM) images were acquired from drop casted samples on mica sheets to study the morphology pattern of these RCM particles. Average height values (H_av_) of a representative sample were 25 ± 5 nm (Fig 3G). Physico-chemical characterizations of Polybees and Lipobees suggest a potential over-edge for Polybees over Lipobees in formulation stability and other prerequisites to make them better agents for systemic application (Table 2).

**Table 2:** Hydrodynamic diameter distribution, anhydrous state particle size, particle height and electrophoretic potential distribution of PRCM, Polybee and LRCM and Lipobee in tabular form.

Nanoparticle	D_av_/DLS (nm)	D_ah_/TEM (nm)	H_av_/AFM (nm)	ζ/Zeta (mV)
LRCM	23±2	35±5	20±6	-12±1
Lipobee	83±3	25±5	25±7	-10±1
PRCM	25±5	25±7	25±5	-12±1
Polybee	40±8	22±6	26±8	-06±3

**Fig 4:**
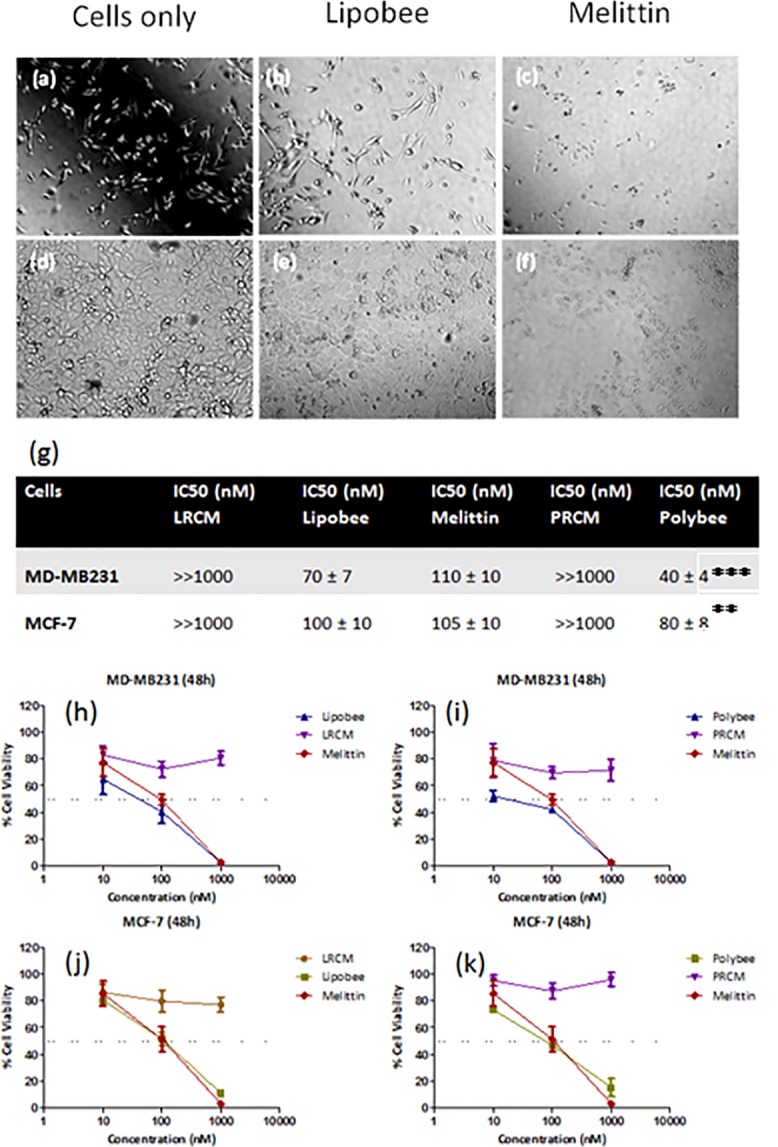
Functional characterization in vitro. Representative bright field images of cell growth density and cancer cell morphology variation for MCF-7 (a-c) and MD-MB231 (d-f) after 48 h of incubation treated with melittin, LRCM and Lipobee, (g) IC50 values for various formulations in tabular form; biostatistical analysis on IC50 values for Polybee respect to melittin representing *** for p value < 0.001 and ** for p value < 0.005 after ONE way ANOVA with Bonferroni post test and (h-i) % cell viability variations by different formulation in MD-MB231 and (j-k) MCF-7 cells for (h-j) polymeric and (i-k) lipidic formulations.

To verify the cancer cell regression affinity of these formulations, cytotoxicity assays were performed. As a model system for *in vitro* cancer culture, we chose estrogen positive (MCF-7) and estrogen negative breast cancer cells (MD-MB231) to evaluate the functional therapeutic potential of Polybees and Lipobees. MCF-7 and MD-MB231 cell lines represent early-stage and invasive human breast cancer cell lines, respectively. Irrespective of the cell line, Polybee showed significantly higher efficacy in comparison to Lipobee and melittin as evident from MTT assays (Fig 4). At the 48h incubation point, in MD-MB231 cells, the IC50 value for Polybee has been found at ca. 40 ± 4 nM compared to ca. 70 ± 7 nM in case of Lipobee and ca. 110 ± 10 M for free melittin; moreover, in MCF-7, IC50 value for Polybee was found to be ca. 80 ± 8 nM compared to ca. 100 ± 10 nM in the case of Lipobee and 105 ± 10 nM for free melittin. Meanwhile, LRCM and PRCM showed IC50 >> 1000 nM irrespective of the cell line, (Fig 4G). For the cells treated with free melittin (100 nM), cell growth density and morphological changes were indicative of cell death, whereas LRCM and PRCM did not alter to any significant level (Fig 4).

**Fig 5:**
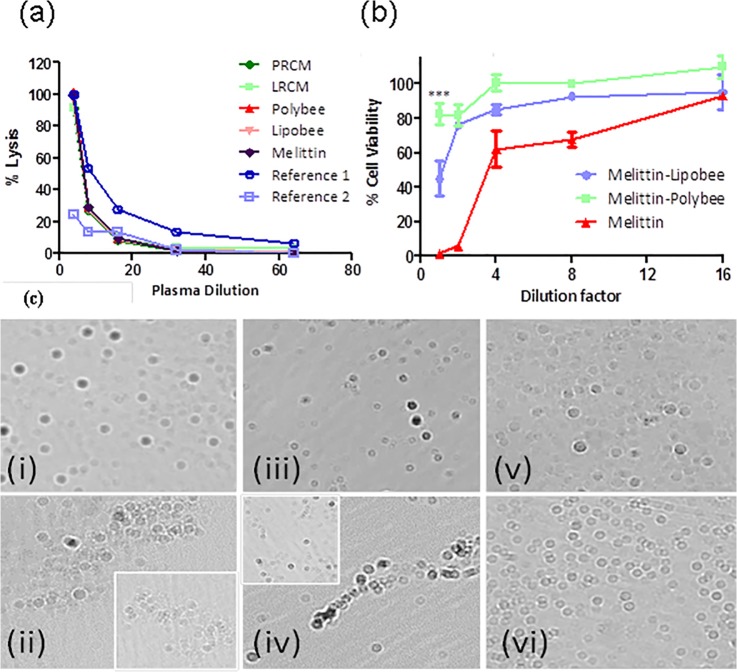
Release mechanism, systemic toxicity and stability studies. (a) Complementing activation and (b) melittin leaching behavior of Lipobee and Polybees. Free melittin, LRCM and PRCM were used as controls; (c) optical microscopy images of blood smear untreated (i) and treated with melittin (1:10) (ii), LRCM (1:10) (iii), Lipobee (1:10) (iv), PRCM (1:10) (v) and polybee (1:10) (vi), respectively, (with 20x magnification). Melittin- and Lipobee-treated pig blood in the severely clumped, morphologically distorted state are shown in (ii) and (iv). Insets in (ii) and (iv) show red blood cell morphology to emphasize other similar morphological patterns throughout the sample.

The CH50 values for all the used formulations were found to be 6 ± 1 for PRCM, LRCM, Polybee, Lipobee and melittin and 8 ± 1 and 3 ± 1 for Reference 1 and Reference 2, respectively (Fig 5A). Although, *in vitro* experiments established Polybee and Lipobee as potent anti-cancer formulations, their presumed behavior for *in vivo* applications still remained unclear. *In vivo* success of such formulations very much depend on two major factors i) neutrality toward blood complement and ii) sustainable passaging of payload through systemic circulation. Our computational studies are indicative of stronger, tighter interaction of melittin with the amphiphilic polymer chains in direct comparison with the lipid, making Polybees; hence, melittin with its rigid core and polymeric shell is a better candidate for *in vivo* application. To confirm this observation experimentally, we explored the complement activation and melittin sustaining ability of these formulations in blood serum. Complementing this system, a group of proteins will activate to lead target cell lysis and facilitate phagocytosis through opsonisation on exposure to solid foreign materials in circulatory systemic fluid. The CH50 assay, which screens the activation of classical complementary pathways found to be sensitive to the reduction, absence and/or inactivity of any component of the pathway. The complementary CH50 assay is based on lysis of sensibilized sheep erythrocytes in the presence of Ca^2+^ and Mg^2+^. When sensibilized sheep erythrocytes are incubated with test serum of different treatments, different levels of haemolysis are achieved. CH50 complement activation assay was performed for all the formulations used here and found to be very inert in activation of complementary proteins, showing no significant change in CH50 values compare to normal complementary level plasma (Reference 1, i.e., 8 ± 1).

The CH50 values for all the used formulations were found to be 6 ± 1 for PRCM, LRCM, Polybee, Lipobee and melittin and 8 ± 1 and 3 ± 1 for Reference 1 and Reference 2, respectively. It indicates that formulation PRCM, LRCM, Polybee, Lipobee and melittin did not induce any complement to any significant level. It supports the feasibility of using these formulations *in vivo* without risk of inducing immune response.

To further assess the benefits of using Polybee over Lipobee for systemic delivery, melittin leaching characteristic of these formulations were evaluated and estimated by performing an MTT assay on MD-MB231 cells. Leached melittin obtained after incubating Polybee and Lipobee with 10% fetal bovine serum (FBS) in the DMEM buffer was used at dilutions 1, 2, 4, 8 and 16 for MTT assays. A known melittin concentration was used as a positive control ranging from 20–1.25 μM. MTT assays exhibited a high amount of melittin leaching from Lipobee causing a high percentage of cell deaths at each dilution compared to cells treated with melittin leaching from Polybee which gives a highly significant bio-statistical significance (p <0.001) at dilution factor 1. On the other hand, at the same dilution, melittin leached out from Polybee, resulting in a significant decline in cell population death with no significant change in cell viability (Fig 5B). These findings indicated that during systemic administration of Lipobee, a high amount of melittin might leach out in the circulatory fluid before reaching the cancer cells thereby causing a significant loss in anti-cancer efficiency.

Polybee nanoparticles have also exhibited noteworthy vigor when admixed with pig blood. A ‘blood smear’ preparation was made to identify any morphological variations in lymphocytes and blood clumping monitored by a clinical optical microscopy technique (conventional light microscopy) under a high power field (Fig 5C). No superficial plodding or morphological vicissitudes in blood cells were observed in fresh pig blood treated with PRCM, LRCM and Polybee (blood: NP = 10:1). However, pig blood treated with free melittin and Lipobee exhibited significant clumping and morphological alterations (Fig 5C, ii and iv). To understand the plausible mechanism of melittin release from Lipobee and Polybee *in vitro*, further studies were conducted. Release of therapeutic agents from nanoparticles has been reported as pH responsive and/or interactive with the anionic layer of the endosomal compartment in cellular systems. These factors can investigate the responsiveness of used nanoparticles for reaching a probable release mechanism. We used this strategy to narrow down our selection of preferential pathways for melittin release from Lipobee and Polybee nanoformulations. Lipobee and Polybee were prepared as discussed earlier followed by incubation at pH 4.6 and in the presence of sodium dodecyl sulphate (SDS, 1 mM) for 2 h. At the end of the incubation period, a hydrodynamic diameter was acquired for different formulations through the use of dynamic light scattering. Sizes at 0 h were considered as control for the experiments (S2 Fig).

DLS results clearly show the variation in size of Lipobee and Polybee only in presence of SDS (5 mM) without any significant effect at a lower pH (S2 Fig). It supports the plausible occurrence of anionic interaction in an endosomal compartment responsible for melittin release from Polybee and Lipobee. Additionally, a higher change in size of Lipobee occurred upon incubation with SDS signifying a higher lipid-surfactant interaction.

Peptide-polynucleotide interactions have always attracted the interest of medicinal chemists. It is of special interest to decipher melittin DNA interactions and understand their role in dissociation from the DNA secondary structure. Gel electrophoresis was performed to enable observation of the changes in electrophoretic mobility patterns of pBR322 incubated with various formulations in the presence (melittin, Polybee and Lipobee) and in the absence of melittin (LRCM and PRCM) as well as various concentrations of free melittin (50–0.0005 μM).

It was found that only free melittin was able to dissociate the plasmid DNA. In turn, the loss of the electrophoresis band was accomplished either by retarding the DNA migration or by expelling the intercalated EtBr (Ethidium bromide) due to major groove binding of melittin in the DNA duplex. Formulations with protected melittin in either of the cases, Lipobee and Polybee, did not influence the DNA bands to any significant extent (S3A Fig).

A further gel electrophoresis investigation showed that ~0.05 μM free melittin was sufficient to start the dissociation of a DNA secondary structure (S3B Fig). The interactions of melittin with DNA in free form signify that a release from Lipobee and Polybee can target genomic DNA, which might extend to the level of hindering the transcription process. However, no interaction can take place if melittin is stably incorporated. Here no significant effect from LRCM and PRCM on DNA mobility showed that these particles do not have an active role to play in DNA interaction and melittin is the only component in Polybee and Lipobee to participate in interaction with DNA duplex.

Proteins are major constituents of blood serum and counter the load vehicle and pharmacoactive agents while delivering through systemic circulations. Any obstruction in normal behavior of such blood serum proteins might lead to harmful consequences to the subject. As a model system of study, we chose fetal bovine serum to establish effects of free melittin and its nanoformulations, Lipobee and Polybees on normal spectroscopic properties. UV absorbance study was performed on 50 μM melittin incubated (free or nanoformulation form) 10% FBS solution. No hypso- or bathochromic shift in UV absorbance pattern was noted from FBS solution but absorbance was increased after incubation with free melittin. A decrease in absorbance was seen in case of Lipobee, which reached a level similar to nontreated FBS when compared to Polybee (S3C Fig). This observation could be explained due to the additional absorbance from added formulations, signifying no specific interaction of melittin in free or nanoformulation form with serum proteins.

**Fig 6:**
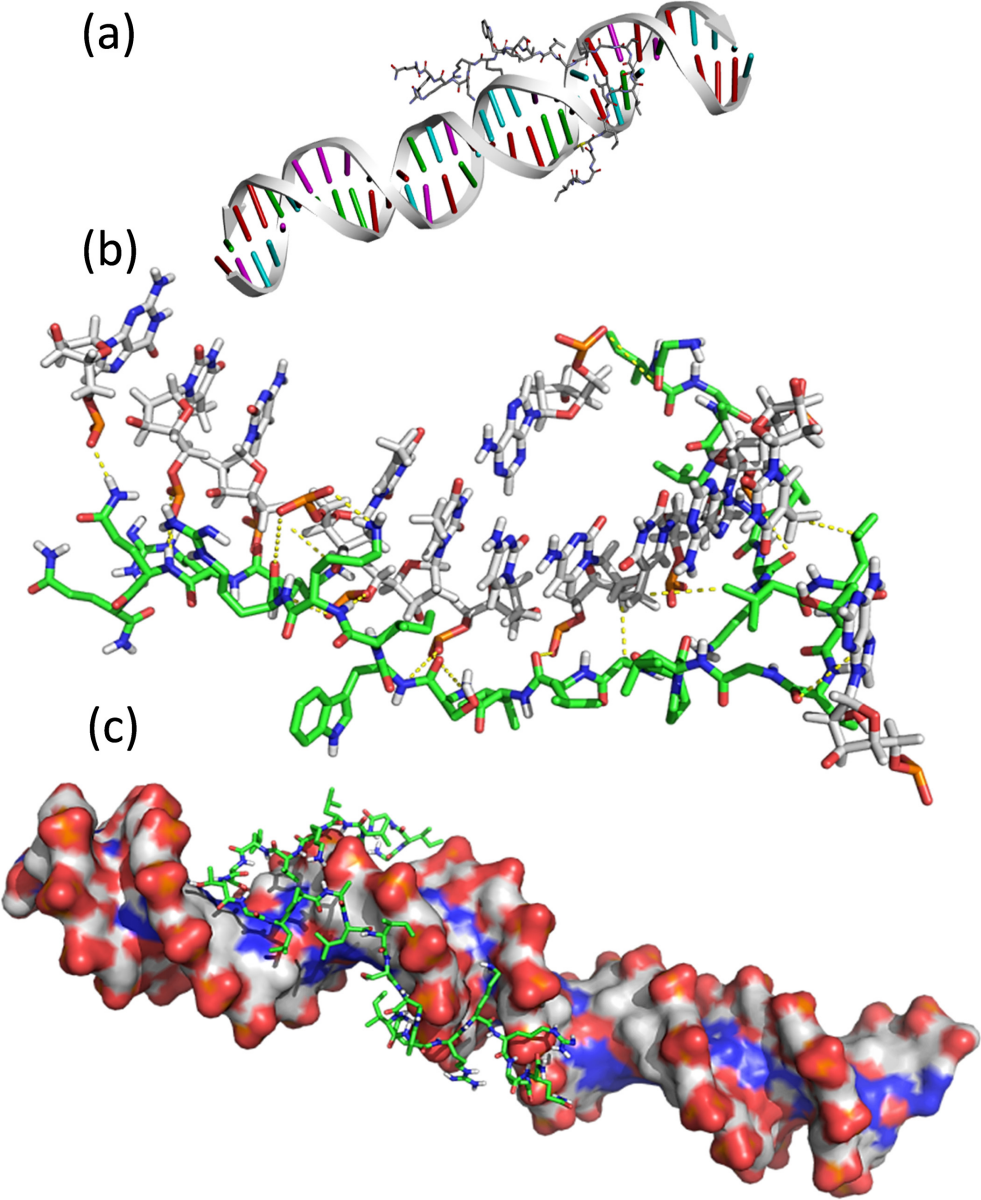
DNA interaction studies. (a) Illustration of docked structure of melittin with DNA. (b) Key interactions of melittin with DNA. Melittin is shown as green links. (c) Molcad surface picture of docked structure.

The fate of released melittin can be rationalized in regards to its interaction with genomic pool cellular components. One among them could have resulted from an interaction of melittin with DNA. To explore the interactions of melittin with DNA in-silico, we performed docking studies of melittin into double stranded DNA (Fig 6). It has been found that melittin intertwines well and remains within a close proximity of DNA structures, forming 17 H-bond interactions and 3 hydrophobic interactions with phosphate groups and base pairings of DNA. It has also been noticed that nitrogen and oxygen in both backbones and side chains formed an H bond only with oxygen of phosphate groups, not with nitrogen and oxygen in paired bases. However, these strong H-bond interactions changed the conformations of paired bases, making the H-bond distance between paired bases mostly enlarged (See S1 Table). The low p-value (0.000415) implied that the conformation of bases of DNA was statistically different under the interactions of melittin. Side chains of Leu and Ala formed hydrophobic interactions with thymine. Docking energy was also found to be quite low (-12.12 mmol/kcal) due to multi-interactions and stable docked structures.

We have established that PRCMs are optimized assemblies for melittin, which can improve the inhibition of cancer cell growth based on various hydrogen bonding connections, which originate between the polymer molecule PS_67_-*b*-PAA_27_ and melittin. Further studies with various fragments of melittin and its sequences reveal the variation in both docking energies and in the resultant final outcomes of the interactions. These measures could be extrapolated for other peptides too, interacting in totally different ways resulting in different morphology, size, stability and extent of delivering the peptide. These properties can be optimized in many ways whereby final outcomes can show results similar to those discussed here.

## Methods

Polyoxyethylene (20) cetyl ether and poly(styrene)-block-poly(acrylic acid) (PS_67_-*b*-PAA_27_) were obtained from Sigma Life Sciences (St. Louis, MO, U.S.A). Tetrahydrofuran (THF) was obtained from Avantor Performance Materials (Center Valley, PA, U.S.A.). Bee venom peptide melittin was obtained from Sigma Aldrich, Inc. (St. Louis, MO, USA). The hydrodynamic diameter was measured on a Malvern Zeta sizer machine equipped with a 633-nm laser. Zeta potential measurement was performed on a Malvern Zeta sizer instrument. Atomic force microscopy was performed on MFP-3D AFM from Asylum Research using Igor Pro software. The TEM images were acquired on JEOL 2100 Cryo TEM machine and imaged by Gatan UltraScan 2kx2k CCD.

### Computational Modeling

Mellitin, lethicin PC and and PS_67_-*b*-PAA_27_ polymer were built using a SKETCH module in Sybyl-X 2.0. Structural energy minimization was performed with the Tripos force field until a gradient convergence of 0.05 kcal/mol was achieved. The NB Cutoff was set to 8.00. Distance was set to the dielectric function, and the diaelectric constant was set to 1.00. MOE-Dock was used for the docking process with a MOE 2013.08 program. InducedFit was chosen as the docking protocol. The selected active sites were whole target molecules, which are PS_67_-*b*-PAA_27_ polymer and Lethicin PC, respectively. Triangle Matcher, which generated poses by aligning ligand triplets of atoms on triplets of alpha spheres, was used as the placement method with default settings.

**Fig 7:**
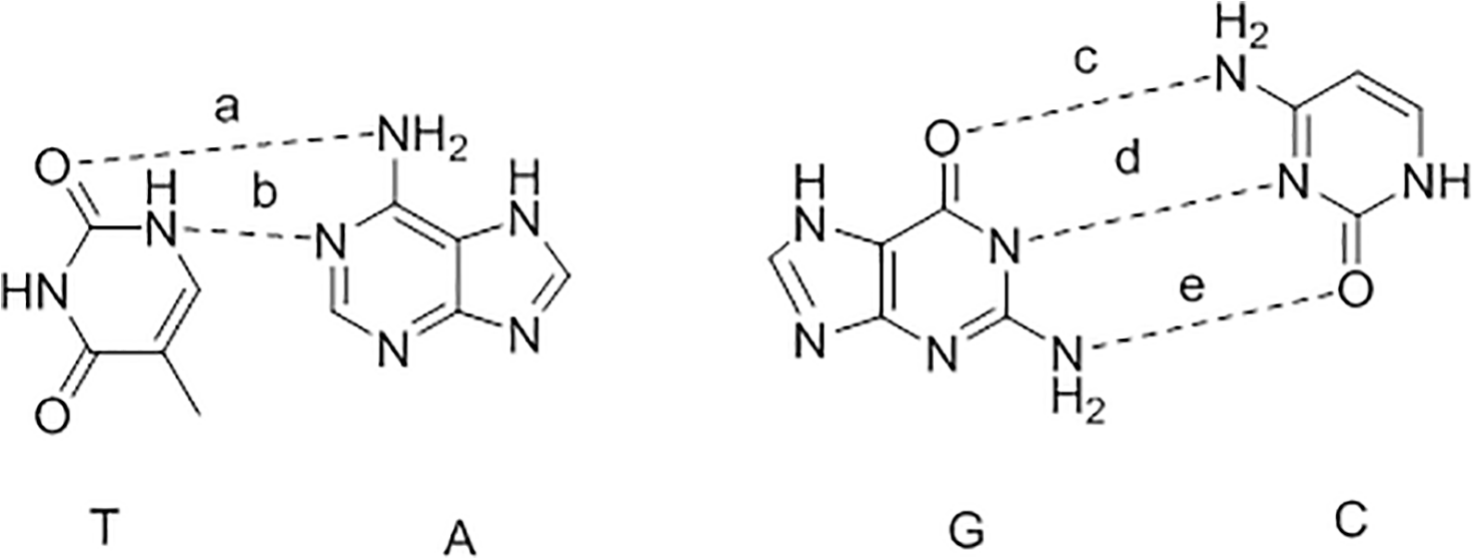


London dG was chosen as the scoring function. The free energy change upon binding of ligand to a receptor was calculated as Eq.1.

$$\Delta G=c+E_{flex}+\sum_{h-bonds}\;c_{HB}f_{HB}+\sum_{m-lig}\;c_{M}f_{M}+\;\sum_{atoms\;i}\;\Delta D_{i}$$ (1)

where c is average entropy loss/gain due to rotational/translational motion; Eflex is entropy loss due to conformational flexibility; cHB is H-bond maximum energy, and H-bond fHB measures geometric imperfections. cM is metal ligation maximum energy. Metal ligation fM measures geometric imperfections; ΔDi estimates the desolvation energy of each atom where i.5 poses were retained for the docking results analysis. The 1st pose with best score, i.e., lowest S value, was chosen as the docking pose. H-bond distance changes between bases of DNA after docking with melittin. (Fig 7)

### Materials Preparation

#### Preparation of PRCM

Polyethylene glycol cetyl ether (2 mg) was melted at 65°C for 5 min followed by the dropwise addition of 2 ml of water (approximately 1 drop/sec). The solution was allowed to stir for 20 min at 1150 rpm. Simultaneously, a solution of poly(styrene) 67-block-poly (acrylic acid) 27 (PS_67_-*b*-PAA_27_, Mn 1,600–1,950 (poly(acrylic acid)), Mn 6,500–7,000 (polystyrene), Mn 8,100–9,100, average Mn 8,700, mp: 192–197°C; Mw/Mn = 1.2) was prepared by adding 2 mg of the amphiphilic polymer and 1 ml of tetrahydrofuran (THF) to a glass vial. After the polyethylene glycol cetyl ether miceller suspension was stirred for 20 min, 250 μl of PS67 PS_67_-*b*-PAA_27_/THF solution was added drop-wise (approximately 1 drop/10 sec) to the solution. The solution was left for stirring overnight to allow THF evaporation. At the end of the procedure, volume was increased up to 2 ml with autoclaved nanopure water (0.2 μM). The suspension was further allowed to stir for 10 min at room temperature. The hydrodynamic diameter of prepared nanoparticles was measured using a nano series Zetasizer. Finally, the suspension was stored at 4°C overnight for curing the core of the particle and the particle size measurement was repeated. The nano particles were purified by dialysis against nanopure (0.2 μM) water using a 20,000 Da MWCO cellulose membrane for a prolonged period of time and then passed through a 0.45 μm Acrodisc Syringe filter. The nanoparticles were stored under argon atmosphere typically at 4°C in order to prevent any bacterial growth.

$$DLS\;(D_{av})/nm\;=25\;\pm \;5\;nm;\;TEM\;(D_{ah})/nm\;=\;25\;\pm \;7\;nm\;AFM\;(H_{av})/nm=25\;\pm \;5\;nm;\;Zeta\;(\zeta )/mV\;=\;-12\;\pm \;1\;mV.$$

#### Preparation of LRCM

Polyethylene glycol cetyl ether (1 mg) was melted at 65°C for 5 min and 1.33 mg of lecithin PC was added. The mixture was allowed to stir for 20 min at 1150 rpm. Simultaneously, 1 ml of water was warmed up at 60°C and added to the mixture dropwise (approximately 1 drop/sec). The solution was left for stirring for 30 min and subsequently cooled down at room temperature. The hydrodynamic diameter of as-synthesized nanoparticles was measured using a nano series Zetasizer. Finally, the suspension was stored at 4°C overnight to allow the core of the particle to be cured and the particle size measurement was repeated for 5 days. The nanoparticles were purified by dialysis against nanopure (0.2μM) water using a 20,000 Da MWCO cellulose membrane for a prolonged period of time and then passed through a 0.45 μm Acrodisc Syringe filter. The nanoparticles were stored under argon atmosphere typically at 4°C in order to prevent any bacterial growth and characterized by various physico-chemical techniques [14, 15].

$$DLS\;(D_{av})/nm\;=\;23\;\pm \;2\;nm;\;TEM\;(D_{ah})/nm\;=\;35\;\pm \;5\;nm\;AFM\;(H_{av})/nm=\;20\;\pm \;6\;nm;\;Zeta\;(\zeta )/mV\;=\;-12\;\pm \;1\;mV.$$

#### Preparation of Polybee and Lipobee

5 mM of Melittin solution was made from a dilution of 20 mM of melittin aqueous solution in 100 mM KCL in nanopure water. 40 μl of 250 μM melittin and 750 μl of PRCMs and LRCMs (as synthesized above) were mixed to make the 5 μM melittin containing Polybee and Lipobee, respectively. The mixed suspension was subsequently vortexed and incubated at room temperature for 30 min before storing at 4°C. The nanoparticles were purified by dialysis against nanopure (0.2 μM) water using a 20,000 Da MWCO cellulose membrane for a prolonged period of time. Melittin loaded formulations were characterized by various physic-chemical techniques [14, 15].

$$\text{Polybee}:\;DLS\;(D_{av})/nm\;=\;40\;\pm \;8\;nm;\;TEM\;(D_{ah})/nm\;=\;22\;\pm \;6\;nm\;AFM\;(H_{av})/nm\;=\;26\;\pm \;8\;nm;\;Zeta\;(\zeta )/mV\;=\;-6\;\pm \;1\;mV.$$

$$\text{Lipobee}:\;DLS\;(D_{av})/nm\;=\;83\;\pm \;3\;nm;\;TEM\;(D_{ah})/nm\;=\;25\;\pm \;5\;nm\;AFM\;(H_{av})/nm\;=\;25\;\pm \;7\;nm;\;Zeta\;(\zeta )/mV\;=\;-10\;\pm \;1\;mV.$$

#### Dynamic light scattering measurements

Hydrodynamic diameter distribution and distribution averages for Polybee, Lipobee, PRCM and LRCMs in aqueous solutions were determined using a Malvern Zetasizer nano series–Nano ZS90. Scattered light was collected at a fixed angle of 90°. A photomultiplier aperture of 400 mm was used, and the incident laser power was adjusted to obtain a photon counting rate between 200 and 300 kcps. Only measurements for which the measured and calculated baselines of the intensity autocorrelation function agreed to within +0.1% were used to calculate nanoparticle hydrodynamic diameter values. The measurements for the particles were made at 0 h, 24 h, 48 h, 72 h, 96 h, and 120 hr after synthesis to evaluate the stability. All determinations were made in multiples of five consecutive measurements.

#### Zeta potential measurements

Zeta potential (ζ) values for Polybee, Lipobee, PRCMs and LRCMs were determined with a nano-series Malvern Zetasizer zeta potential analyzer. Measurements were made following dialysis (MWCO 20 kDa dialysis tubing, Spectrum Laboratories, Rancho Dominguez, CA) of nanoparticle suspensions into water. Data were acquired in the phase analysis light scattering (PALS) mode following solution equilibration at 25°C. Calculation of ζ from the measured nanoparticle electrophoretic mobility (μ) employed the Smoluchowski equation: μ = εζ/η, where ε and η are the dielectric constant and the absolute viscosity of the medium, respectively. Measurements of ζ were reproducible to within ±5 mV of the mean value given by 20 determinations of 10 data accumulations

#### Atomic force microscopy measurements (AFM)

Atomic force microscopy (AFM) was performed to observe the morphological topography in PRCM and LRCM structures. The samples were drop cast onto freshly cleaved mica sheets and air-dried for 24 h. Topographic imaging of all the formulations was obtained by operating the AFM in a tapping mode with an Asylum Cypher AFM instrument. The average particle height (H_av_) values and standard deviations were generated from the analyses of a minimum of 50 particles from three micrographs. Analysis of the AFM images was processed using ImageJ.

#### Transmission electron microscopy measurements (TEM)

The Transmission electron microscopy (TEM) was performed on Polybee, Lipobee, PRCMs and LRCMs to evaluate their morphologies. Imaging was performed on samples prepared on copper grids coated with a formvar plastic and then coated with carbon for stability followed by negative staining with 7% Uranyl acetate.

#### Human transformed cancer cell culture

MD-MB231 cells (ER (-) breast cancer cells) and MCF-7 cells (ER (+) breast cancer cells) were cultured in Dulbecco’s Modified Eagle’s Medium (DMEM; Sigma) supplemented with 10% fetal bovine serum (FBS) in T25 culture flasks (Cellstar; Germany) and were incubated at 37°C in a 99% humidified atmosphere containing 5% CO2. Cells were regularly passaged by trypsinization with 0.1% trypsin (EDTA 0.02%, dextrose 0.05%, and trypsin 0.1%) in DPBS (pH 7.4). Non-synchronized cells were used for all the experiments.

#### MTT Assay

The cell viability of Polybee, Lipobee, PRCM, LRCM and melittin formulations in used MD-MB231 and MCF-7 cells were investigated by using 3-(4,5-dimethylthiazole-2-yl)-2,5-diphenyltetrazolium bromide (MTT) [16] reduction assay in presence of 10% FBS in antibiotic free media. Experiments were performed in 96 well plates (Cellstar; Germany) growing 8,000 cells per well 24 h before treatments. Experiments were performed for various concentrations of melittin ranging from 10 to 1000 nM present in free or Polybee and Lipobee forms while the same volume of LRCM and PRCM was used as negative controls. Cells were incubated for 48 h before performing the MTT assay. After incubation period, cells were imaged for investigating growth density and morphology variations. Cells were further treated with MTT as 20 μl (5 mg/mL) per well and further incubated for 5 h. At the end of the incubation, the entire medium was removed from wells and 200 μL of DMSO was added to dissolve blue colored formazan crystals. The percentage cell viability was obtained from a plate reader and was calculated using the formula % Viability = {[A630(treated cells)- (background)]/[A630(untreated cells)-background]}x100.

#### Plausible mechanism of melittin release from Lipobee and Polybee in vitro

To establish the possible pathway of melittin release from Lipobee and Polybee formulations, experiments were performed by incubating the formulations with SDS (5 mM) at pH 4.6 for 2 h before acquiring their hydrodynamic diameter [17, 18]. A significant change in hydrous diameter was achieved by interaction with SDS and by not lowering pH to 4.6, which revealed the repackaging of structural components only in presence of anionic assemblies, during which melittin might get released.

#### Blood-smear experiment

A single smear was made per slide by putting a drop of fresh pig blood on the slide (near the end). The drop was spread by using another slide (“spreader”), placing the spreader at a 45° angle and backing into the drop of blood [19, 20]. The spreader catches the drop and it spreads by capillary action along its edge. Smear is allowed air dry for 10 min and cover-slip before placed directly on the microscope and observed under 40 x magnifications. A ratio of 1:9 Polybee, Lipobee, PRCM, LRCMs and melittin (10 μl; melittin conc. 50 nM) and pig blood was used for preparation of the smear.

#### Melittin-DNA interaction and dissociation of primary and secondary structure

The most favorable interactions between amino acids and nucleobases are toward arginine and lysine with guanine and also for lysine with thymine. These preferences could explain partial charge interactions between amino acid side chains and base functionality in the major groove [21]. It is interesting that electrostatic interactions are of critical importance but an additional contribution might come from the flexible side chain interactions viz. hydrogen bonding and hydrophobic interaction, which might also play significant role. Interactions between amino acid residues and DNA bases are introduced by H-bonding or hydrophobic packing interactions in the major groove. Amino acid residues Glu and Asp are known to have H-bond acceptors but not donors, and they only interact with C or A bases. Ser, Cys, and Thr play dual roles as both donor and acceptor, and Arg and Lys play H-bond donor but not acceptor. Hydrophobic interaction in the major groove of DNA is the single methyl group of the T base. Hydrophobic interactions can occur between Ala, Val, Ile, Leu, Met, Phe, Tyr, Trp and Thr with three methyl groups on T base. Similarly two ring CH groups of the C bases can also interact with hydrophobic residues; however, interaction with the hydrogen atoms of C will not be as strong as with the methyl group of the thymine. On the other side, Ala appears to be insufficiently hydrophobic to contact the C base.

Keeping these possible interactions in mind, we want to explore interaction patterns and possible dissociation in the secondary structure of plasmid DNA, as a model duplex system with melittin [22]. An experiment was performed by incubating plasmid DNA pBR322 with melittin in free or Lipobee and Polybee forms while LRCM and PRCM were used as negative controls. Another experiment was performed on 1% agarose gel in TAE buffer. The pBR322 plasmid DNA (0.3 μg/cocktail) was used to mix with melittin, Polybee and Lipobee (with a melittin concentration of 50 μM). LRCM and PRCM were used with equal volume as in case of Polybee and Lipobee formulations. To know the effect of melittin concentration, another set of cocktails were also prepared using the same amount of DNA per cocktail but with melttin concentration varying as 50, 5, 0.5, 0.05, 0.005 and 0.0005 μM. At the end of 1 h of incubation at room temperature, cocktails were mixed with 6x DNA loading dye and loaded on gel for running electrophoretically. Untreated DNA was run as control for the experiment. After running the gel, it was stained in ethidium bromide (20 mg/100 ml) for 10 min followed by washing for 5 min to remove excess EB staining. Gels were imaged under UV light.

#### Interaction of melittin with serum proteins

To investigate the interaction pattern of melittin and melittin-based Lipobee and Polybees with serum proteins, FBS (fetal bovine serum) was used for the model system [23]. A 10% FBS solution was incubated with 50 μM Melittin in free or Lipobee and Polybee forms. Samples were incubated for 4 h before measuring the UV absorbance.

#### Biostatistical Analysis

To evaluate the extent of improvements in activity of melittin against cancer cell growth when formulated as Polybee, ONE way ANOVA was performed. It was performed on IC50 values with a Bonferroni post test to calculate p values ay 0.001 and 0.005 and represented as *** and ** values next to the values.

## Conclusions

We have established an *in-silico-to-in-vitro* approach to synthesize a well-defined, self-assembled, rigid-cored polymeric (polybee) nano-architecture for controlled delivery of a key component of bee-venom, melittin. A competitive formulation with lipid-encapsulated (Lipobee) rigid cored micelle was synthesized. In a series of sequential experiments, we studied how nanoscale chemistry influences the delivery of venom toxins for cancer regression and helps evade systemic disintegrity and cellular noxiousness. Our experimental and computational results indicated that Polybees were better cancer cell growth inhibitors than Lipobees in two breast cancer cell lines, presumably due to their stable and tighter association with melittin. Our results indicated insignificant to no complementary activation from these particles and free melittin. Studies with sodium dodecyl sulfate and lower pH revealed anionic membrane interactions as probable mechanisms for melittin release from Lipobee and Polybee nanoparticles. UV absorption studies for fetal bovine serum revealed their inactivity against serum proteins while gel electrophoretic assays described very strong interaction of free melittin with plasmid DNA. Extensive molecular docking studies revealed significant changes in base pair H-bonding distances after interaction with melittin. The main reason behind the differential behavior of peptide incorporation in lipid and amphiphilic polymer-based nanoassemblies can be found in the different types of interactions they face. Model lipidic membranes are known to be disrupted after interaction with polypeptides, [24] which are supposed to expand the lipid assembly in case of Lipobee and in turn increase the hydrodynamic diameter by a significant extent. Here none of the components of PRCM and LRCM are designed to respond to lower pH although PS-b-PAA could have improved electrostatic repulsion between the deprotonated PAA chains resulting in the thinning of the vesicle membrane at higher pH only.[25] On the other hand, melittin is known to have different fusion extent to model vesicles of phospahtidyl choline with maximum at 5.1 which could play role in case of cellular interactions of Lipobee and Polybee formulations, [26] but as LRCM and PRCM making major fractions of Lipobee and Polybee, anionic assemblies/membranes are supposed to play major role. On the other hand, we have demonstrated through melittin leaching studies that Polybees are very stable as such and do not change size as much as Lipobee. After release from Polybee particles, melittin could play a significant role in DNA association-dissociation processes, too, which might assist in growth inhibition of the desired cell population. Thus, we conclude that the use of amphiphilic polymer in preparation may provide a better strategy to produce stable formulation of melittin for systemic application compared to lipid-based amphiphiles.

## Supporting Information

S1 Fig Docking poses of peptide fragments showing interactions. Docking poses of 17-residue, 16-residue and 9-residue peptides to PS_67_-*b*-PAA_27_ polymer and lecithin PC. Docking poses of (a) 17-residue peptide, (b) 16-residue peptide, and (c) 9-residue peptide to PS_67_-*b*-PAA_27_ polymer, (d) 17-residue peptide, and (e) 16-residue peptide; (f) 9-residue peptide to lecithin PC. Green links represent peptides. White lines represent PS_67_-*b*-PAA_27_ polymer and lecithin PC.
(TIF)

S2 Fig Responsiveness of the formulation in presence of anionic surfactants and lower pH. Hydrodynamic size distribution of Lipobee (a, c) and Polybee (b, d) suspension in presence of SDS (a and c; 5 mM) and at pH 4.6 incubation (c and d) for 2 h. 1 mL of samples were used for DLS measurements and acquired as multiples of five consecutive runs.
(TIF)

S3 Fig Gel electrophoretic images of plasmid DNA interacted with melittin in free or Lipobee and Polybee forms. (a) 200 ng of plasmid DNA incubated for 60 min with melittin, Lipobee and Polybee formulations with 50 μM melittin and LRCM, PRCM as control; (b) 200 ng of Plasmid DNA incubated with 50–0.0005 μM of free melittin for 60 min before performing gel electrophoresis. (c) UV-spectroscopic behavior of melittin and nanoformulation in presence of fetal bovine serum.
(TIF)

S1 Table H-bond distance of bases before and after docking (T test: p value = 0.000415). (PDF)

S2 Table Docking Scores of 17-, 16- and 9-residue melittin fragments to Polybee and Lipobee system. (PDF)

## References

[1] Keramidas A, Moorhouse AJ, Schofield PR, Barry PH. Ligand-gated ion channels: mechanisms underlying ion selectivity. Prog Biophys Mol Biol. 2004; 86: 161–204. 15288758 10.1016/j.pbiomolbio.2003.09.002

[2] King G. Venoms to Drugs: Translating Venom Peptides into Therapeutics. Aust. Biochem. 2013; 44: 13–16.

[3] Saez NJ, Senff S, Jensen JE, Yan S, Herzig V, Rash LD, et al Spider-Venom Peptides as Therapeutics. Toxins 2010; 2: 2851–2871. 10.3390/toxins2122851 22069579 PMC3153181

[4] Hmed BN, Serria HT, Mounir ZK. Scorpion Peptides: Potential Use for New Drug Development. J Toxicol. 2013: 1–15. 10.1155/2013/958797 PMC3697785 23843786

[5] (a) Garcia ML, Gao Y, McManus OB, Kaczorowski GJ. Potassium channels: from scorpion venoms to high-resolution structure. Toxicon. 2001; 39: 739–748. (b) Han YY, Liu HY, Han DJ, Zong XC, Zhang SQ, Chen YQ. Role of glycosylation in the anticancer activity of antibacterial peptides against breast cancer cells. Biochem Pharmacol. 2013; 86: 1254–1262. (c) Kondo E, Saito K, Tashiro Y, Kamide K, Uno S, Furuya T, et al. Tumour lineage-homing cell-penetrating peptides as anticancer molecular delivery systems. Nature Commun. 2012; 3: 1–13.

[6] Possani LD, Merino E, Corona M, Bolovar F, Becerril B. Peptides and genes coding for scorpion toxins that affect ion-channels. Biochimie. 2000; 82: 861–868. 11086216 10.1016/s0300-9084(00)01167-6

[7] Zuo XP, Ji YH. Molecular mechanism of scorpion neurotoxins acting on sodium channels. Mol Neurobiol. 2004; 30: 265–278. 15655252 10.1385/MN:30:3:265

[8] Elgar D, Plessis JD, Plessis LD. Cysteine-free peptides in scorpion venom: geographical distribution, structure-function relationship and mode of action. Afr J Biotechnol. 2006; 5: 2495–2502.

[9] Misra SK, Ye M, Kim S, Pan D. Highly efficient anti-cancer therapy using scorpion ‘NanoVenin’. Chem Commun. 2014; 50:13220–13223. 10.1039/c4cc04748f 25061638

[10] (a) Guo X, Ma C, Du Q, Wei R, Wang L, Zhou M, et al. Two peptides, TsAP-1 and TsAP-2, from the venom of the Brazilian yellow scorpion, Tityus serrulatus: Evaluation of their antimicrobial and anticancer activities. Biochimie. 2013; 95: 1784–1794. (b) Isa L, Amstad E, Schwenke K, Del Gado E, Ilg P, Kroeger M, et al. Adsorption of core-shell nanoparticles at liquid–liquid interfaces. Soft Matter. 2011; 7: 7663–7675; (c) Padovan-Merhar O, Lara FV, Starr FW. Stability of DNA-linked nanoparticle crystals: Effect of number of strands, core size, and rigidity of strand attachment. J Chem Phy. 2011; 134: 244701/1–7.

[11] SYBYL-X 2.0, Tripos International, 1699 South Hanley Rd., St. Louis, Missouri, 63144, USA.

[12] Molecular Operating Environment (MOE), 2013.08; Chemical Computing Group Inc., 1010 Sherbooke St. West, Suite #910, Montreal, QC, Canada, H3A 2R7, 2013.

[13] Wu J, Eisenberg A. Proton Diffusion across Membranes of Vesicles of Poly(styrene-b-acrylic Acid) Diblock Copolymers. J Am Chem Soc. 2006; 128: 2880–2884. 16506766 10.1021/ja056064x

[14] Elsabahy M, Wooley KL. Design of polymeric nanoparticles for biomedical delivery applications. Chem Soc Rev. 2012; 41: 2545–2561. 10.1039/c2cs15327k 22334259 PMC3299918

[15] Kim B, Schmieder AH, Stacy AJ, Williams TA, Pan D. Sensitive Biological Detection with a Soluble and Stable Polymeric Paramagnetic Nanocluster. J Am Chem Soc. 2012; 134: 10377–10380. 10.1021/ja3040366 22693958 PMC3397310

[16] Fischer D, Bieber T, Li Y, Elsasser HP, Kissel T. A Novel Non-Viral Vector for DNA Delivery Based on Low Molecular Weight, Branched Polyethylenimine: Effect of Molecular Weight on Transfection Efficiency and Cytotoxicity. Pharm Res. 1999; 16: 1273–1279. 10468031 10.1023/a:1014861900478

[17] Chen Y, Dong CM. pH-Sensitive Supramolecular Polypeptide-Based Micelles and Reverse Micelles Mediated by Hydrogen-Bonding Interactions or Host-Guest Chemistry: Characterization and In Vitro Controlled Drug Release, J Phys Chem B. 2010; 114: 7461–7468. 10.1021/jp100399d 20469900

[18] Bhat PA, Rather GM, Dar AA. Effect of Surfactant Mixing on Partitioning of Model Hydrophobic Drug, Naproxen, between Aqueous and Micellar Phases. J Phys Chem B. 2009; 113: 997–1006. 10.1021/jp807229c 19123827

[19] Pan D, Williams TA, Senpan A, Stacy AJ, Scott MJ, Gaffneya PJ, et al Detecting Vascular Biosignatures with a Colloidal, Radio-Opaque Polymeric Nanoparticle. J Am Chem Soc. 2009; 131: 15522–15527. 10.1021/ja906797z 19795893 PMC2826244

[20] Wheater PR, Burkitt HG, Daniels VG. Functional Histology: A text and colour Longman Group; UK: 1987; p. 407–408.

[21] Suzuki M. A framework for the DNA–protein recognition code of the probe helix in transcription factors: the chemical and stereochemical rules. Structure 1994; 2: 317–326. 8087558 10.1016/s0969-2126(00)00033-2

[22] Yadav S, Mahato M, Pathak R, Jha D, Kumar B, Deka SR, et al Multifunctional self-assembled cationic peptide nanostructures efficiently carry plasmid DNA in vitro and exhibit antimicrobial activity with minimal toxicity. J Mater Chem B. 2014; 2: 4848–4861. 10.1039/c4tb00657g 32261776

[23] Yang Y, Yang Y, Xie X, Cai X, Zhang H, Gong W, et al PEGylated liposomes with NGR ligand and heat-activable cell-penetrating peptide–doxorubicin conjugate for tumor-specific therapy. Biomaterials 2014; 35: 4368–4381. 10.1016/j.biomaterials.2014.01.076 24565519

[24] Cao P, Abedini A, Wang H, Tu L-H, Zhang X, Schmidt AM, et al Islet amyloid polypeptide toxicity and membrane interactions. PNAS 2013; 110: 19279–19284. 10.1073/pnas.1305517110 24218607 PMC3845181

[25] Chen Q, Vancso GJ. pH dependent elasticity of polystyrene-block-poly(acrylic acid) vesicle shell membranes by atomic force microscopy. Macromol Rapid Commun. 2011; 32:1704–1709. 10.1002/marc.201100332 21994204

[26] Murata M, Nagayama K, Ohnishi S. Membrane fusion activity of succinylated melittin is triggered by protonation of its carboxyl groups. Biochemistry 1987; 26:4056–4062. 2820482 10.1021/bi00387a047

