# Supplementary figures and images for: Defined Nanoscale Chemistry Influences Delivery of Peptido-Toxins for Cancer Therapy
Source: PLoS One. 2015 Jun 1;10(6):e0125908. doi: 10.1371/journal.pone.0125908 (PMC4452514; doi:10.1371/journal.pone.0125908)

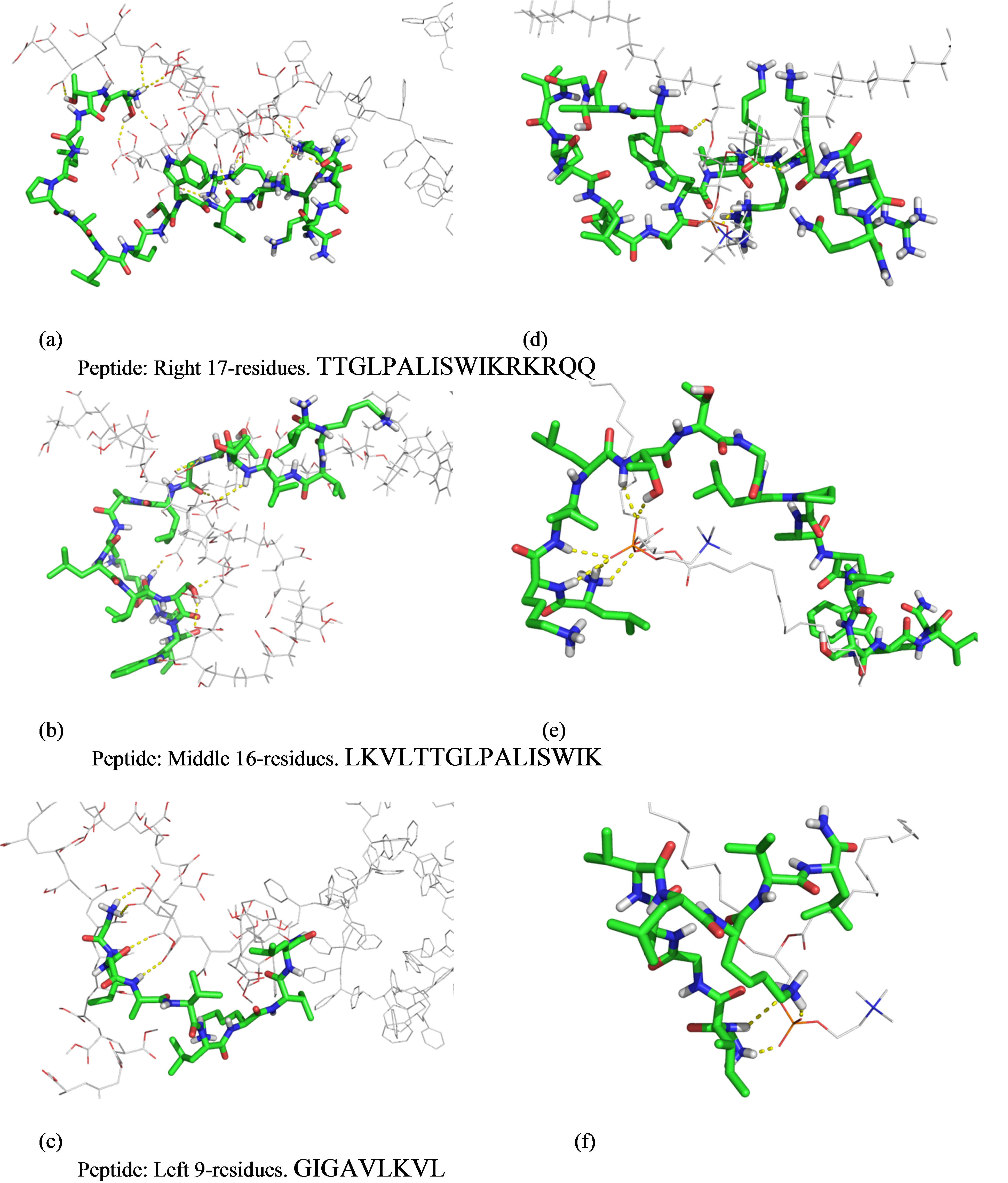

Supplement: S1 Fig — Docking poses of 17-residue, 16-residue and 9-residue peptides to PS67-b-PAA27 polymer and lecithin PC. Docking poses of (a) 17-residue peptide, (b) 16-residue peptide, and (c) 9-residue peptide to PS67-b-PAA27 polymer, (d) 17-residue peptide, and (e) 16-residue peptide; (f) 9-residue peptide to lecithin PC. Green links represent peptides. White lines represent PS67-b-PAA27 polymer and lecithin PC. (TIF) [file pone.0125908.s001.tif]

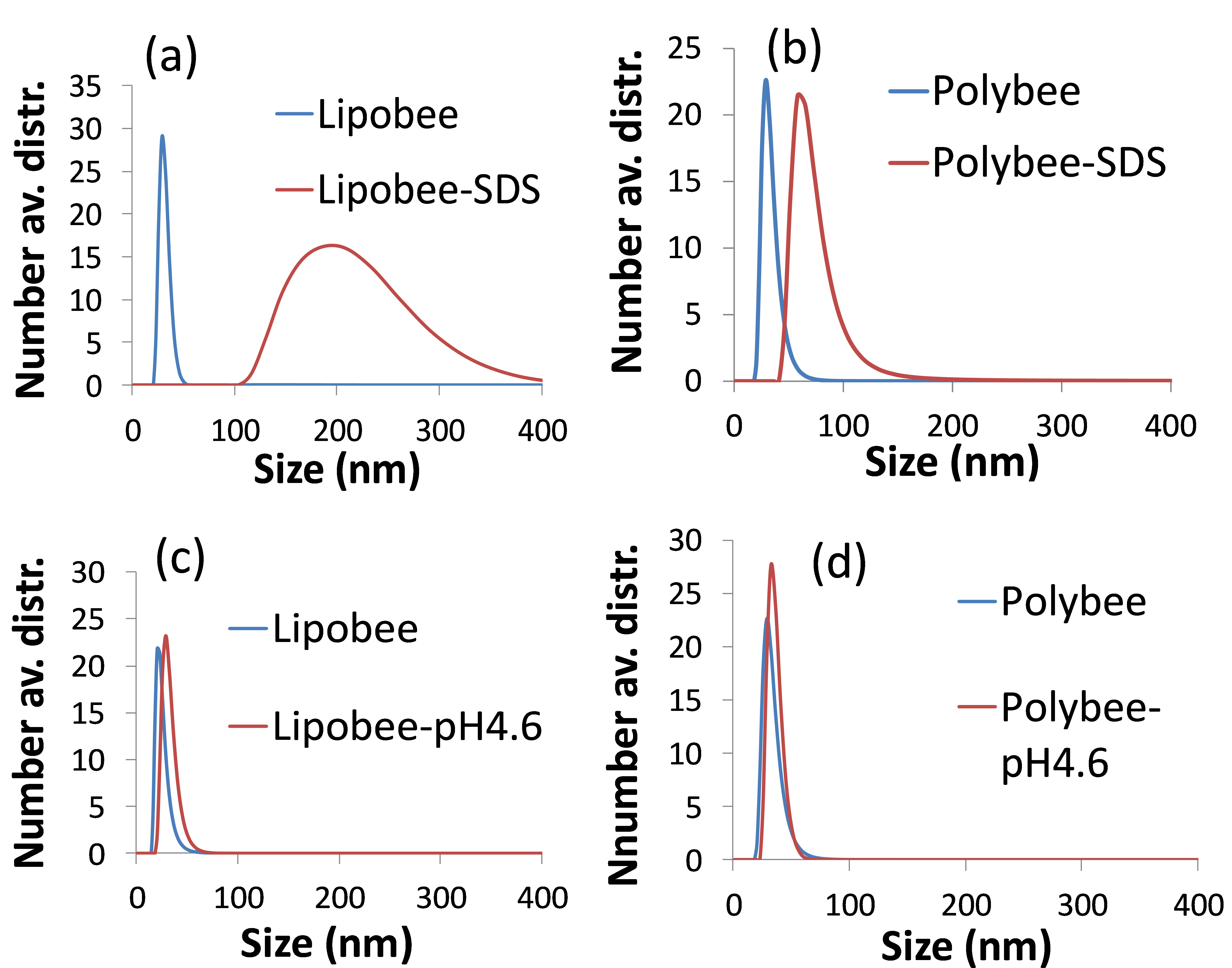

Supplement: S2 Fig — Hydrodynamic size distribution of Lipobee (a, c) and Polybee (b, d) suspension in presence of SDS (a and c; 5 mM) and at pH 4.6 incubation (c and d) for 2 h. 1 mL of samples were used for DLS measurements and acquired as multiples of five consecutive runs. (TIF) [file pone.0125908.s002.tif]

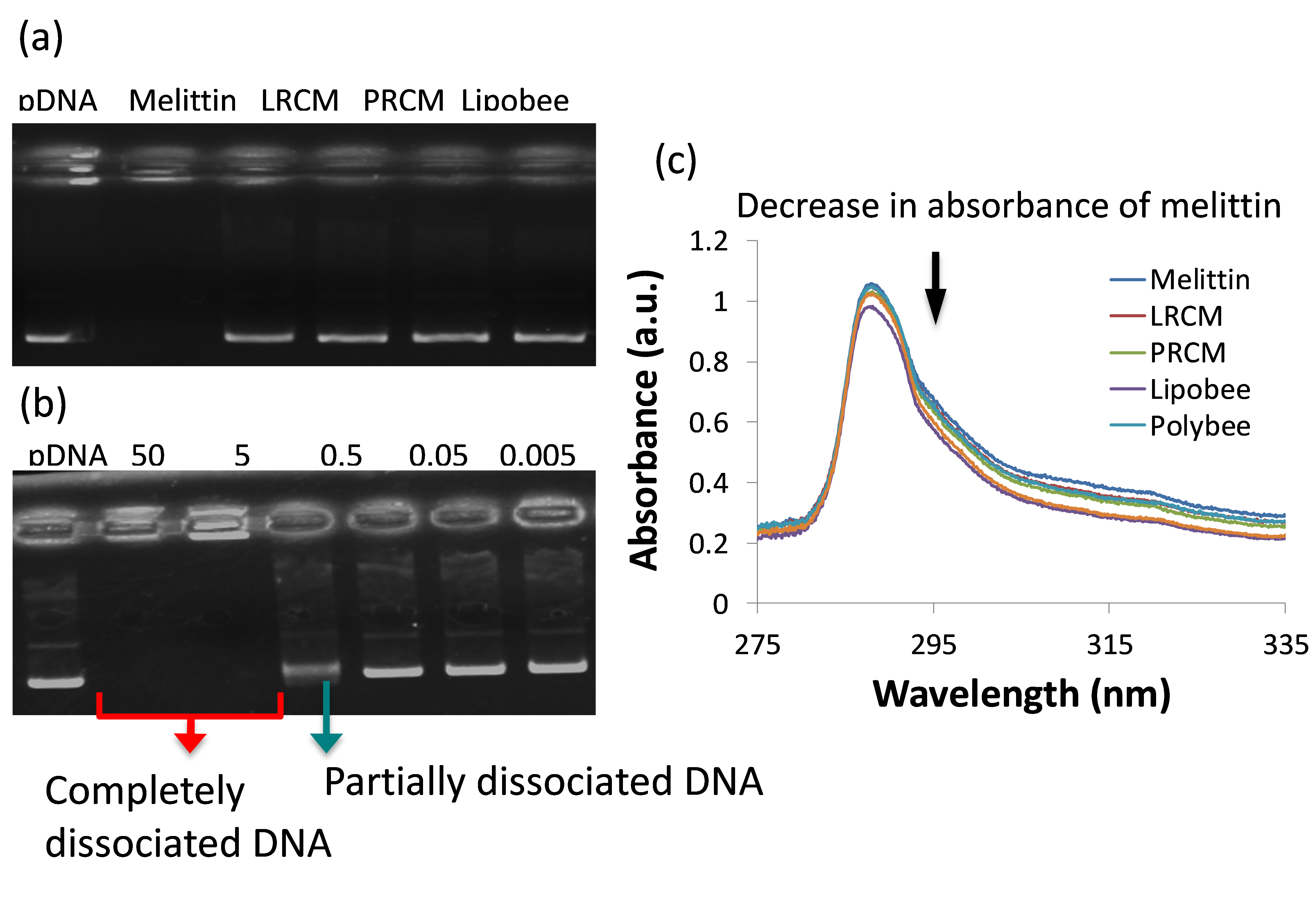

Supplement: S3 Fig — (a) 200 ng of plasmid DNA incubated for 60 min with melittin, Lipobee and Polybee formulations with 50 μM melittin and LRCM, PRCM as control; (b) 200 ng of Plasmid DNA incubated with 50–0.0005 μM of free melittin for 60 min before performing gel electrophoresis. (c) UV-spectroscopic behavior of melittin and nanoformulation in presence of fetal bovine serum. (TIF) [file pone.0125908.s003.tif]
