# Supplementary material for: Defined Nanoscale Chemistry Influences Delivery of Peptido-Toxins for Cancer Therapy
Source: PLoS One. 2015 Jun 1;10(6):e0125908. doi: 10.1371/journal.pone.0125908 (PMC4452514; doi:10.1371/journal.pone.0125908)
Supplement: S1 Table — (PDF) [file pone.0125908.s004.pdf]

No	Strand 1 Base	interactions	Before	After	**Change**	Strand 2 Base
1	A	a	3.2	3.2	**0**	T
		b	3.0	3.0	**0**	
2	A	a	2.8	3.0	**0.2**	T
		b	2.7	2.8	**0.1**	
3	A	a	3.3	3.2	**-0.1**	T
		b	2.7	2.8	**0.1**	
4	T	a	3.1	3.2	**0.1**	A
		b	2.8	2.9	**0.1**	
5	G	c	2.8	2.9	**0.1**	C
		d	2.8	2.9	**0.1**	
		e	2.6	2.9	**0.3**	
6	A	a	3.0	3.1	**0.1**	T
		b	3.0	3.0	**0**	
7	C	c	2.8	2.9	**0.1**	G
		d	2.8	2.9	**0.1**	
		e	2.7	2.9	**0.2**	
8	A	a	2.8	2.9	**0.1**	T
		b	2.9	2.9	**0**	
9	T	a	3.0	3.0	**0**	A
		b	3.0	2.9	**-0.1**	
10	A	a	2.8	2.9	**0.1**	T
		b	2.7	2.8	**0.1**	
11	G	c	2.8	3.0	**0.2**	C
		d	2.7	2.9	**0.2**	
		e	2.6	2.8	**0.2**	
12	G	c	2.9	3.0	**0.1**	C
		d	2.8	3.0	**0.2**	
		e	2.6	2.9	**0.3**	
13	A	a	2.5	2.8	**0.3**	T
		b	2.9	3.0	**0.1**	
14	A	a	3.1	2.9	**-0.2**	T
		b	2.9	2.8	**-0.1**	
15	A	a	3.0	3.1	**0.1**	T
		b	3.1	3.0	**-0.1**	
16	A	a	3.2	2.9	**-0.3**	T
		b	2.9	2.7	**-0.2**	
17	C	c	2.8	3.0	**0.2**	G
		d	2.9	3.0	**0.1**	
		e	2.9	3.0	**0.1**	
18	T	a	3.1	3.2	**0.1**	A
		b	2.9	2.9	**0**	
19	G	c	3.4	3.1	**-0.3**	C
		d	3.1	3.0	**-0.1**	
		e	2.8	3.0	**0.2**	
20	A	a	2.8	3.0	**0.2**	T
		b	2.8	2.9	**0.1**	
21	A	a	2.9	3.0	**0.1**	T
		b	2.6	2.8	**0.2**	
22	A	a	3.3	3.2	**-0.1**	T
		b	3.3	3.2	**-0.1**	
23	G	c	2.7	3.0	**0.3**	C
		d	2.7	2.9	**0.2**	
		e	2.6	2.8	**0.2**	

24	G	c	2.6	3.0	**0.4**	C
		d	2.8	3.0	**0.2**	
		e	2.8	3.1	**0.3**	
25	G	c	2.6	2.9	**0.3**	C
		d	2.7	2.9	**0.2**	
		e	2.7	3.0	**0.3**	
26	A	a	2.8	2.9	**0.1**	T
		b	2.8	2.9	**0.1**	
27	G	c	2.5	2.8	**0.3**	C
		d	2.9	3.1	**0.2**	
		e	3.2	3.3	**0.1**	
28	A	a	2.8	3.0	**0.2**	T
		b	2.8	2.9	**0.1**	
29	A	a	2.7	2.8	**0.1**	T
		b	2.5	2.7	**0.2**	
30	G	c	3.0	3.2	**0.2**	C
		d	3.1	3.2	**0.1**	
		e	3.0	3.1	**0.1**	
