# Supplementary material for: Defined Nanoscale Chemistry Influences Delivery of Peptido-Toxins for Cancer Therapy
Source: PLoS One. 2015 Jun 1;10(6):e0125908. doi: 10.1371/journal.pone.0125908 (PMC4452514; doi:10.1371/journal.pone.0125908)
Supplement: S2 Table — (PDF) [file pone.0125908.s005.pdf]

**Melittin Fragments**	**Scores docked to polybee system**	**Scores docked to lipobee system**
17-Residues	-9.80	-6.70
16-Residues	-8.68	-6.08
9-Residues	-6.17	-4.88
